# Self-Medication in University Students from the City of Mansoura, Egypt

**DOI:** 10.1155/2017/9145193

**Published:** 2017-04-05

**Authors:** R. M. Helal, H. S. Abou-ElWafa

**Affiliations:** Public Health and Community Medicine Department, Faculty of Medicine, Mansoura University, Mansoura, Egypt

## Abstract

*Background*. Self-medication is a common practice in developed and developing countries.* Objectives*. To explore the prevalence of self-medication practices among university students, probable reasons, symptoms requiring self-medication, and sources of advice.* Methods*. A descriptive cross-sectional study was carried out in Mansoura University, Egypt, and included 1st and last year students of both medical and nonmedical faculties.* Results*. Prevalence of self-medication was 62.9%. Younger age, female, medical, and ever-married students and those having home pharmacy tended to self-medicate more than their peers with significant difference between them. Being medical student, being from urban area, having good current health condition, being careless about health, and having drugs stored at home pharmacy were independently associated with the likelihood of self-medicating.* Conclusion*. Prevalence of self-medication among university students is high which constitutes a health problem that needs intervention.

## 1. Introduction

Self-medication is defined as getting and consuming drug without the guidance of physician for either diagnosis, treatment, or supervision of the treatment [1] generally involving over-the-counter (OTC) medications but also including prescription-only medicines (POM) [2], at the same time it includes buying drugs by reutilizing/resubmitting a previous prescription, taking medicines on advice of relative or others, or consuming leftover medicines already available at home [3].

Self-medication with OTC medications is a worldwide public health problem [4] and is more experienced in developing countries [5]. Self-medication patterns vary among different populations and are influenced by various features, such as age, gender, income and expenditure, self-care orientation, educational level, medical knowledge, satisfaction, and nonseriousness of illnesses [6, 7].

Studies revealed that self-medication represents a common problem among university students [3, 8, 9]. Media exposure and the increase of pharmaceuticals advertisement pose a larger threat to this population as it was found that majority of college students used at least one of the advertised products, without discussing it with their physicians [10]. Other reasons for self-medication among university students were their previous experiences, advice of family or friends, their health problems being considered as too trivial, time saving, nonavailability of transport, convenience, ability to self-manage the symptoms, urgency of the problem, doctor that was not available, and having sufficient information [9, 11, 12]. Lack of time, low cost consultation, and trust in medical doctor were reported as main reasons in other studies [8, 13].

Self-medication when practiced correctly reduces the load on medical services, reduces the time spent in waiting to see the physician, and saves cost especially in economically deprived countries with limited healthcare resources [14].

However, it has been found that self-medication can slip towards self-medication with prescription medications and/or improper drug use such as misdiagnosis, low or high doses, and/or treatment duration. Such practices may result in irrational drug use [15], delayed seeking of medical advice, and increased side effects and increase in pathogens resistance that result in wasting of resources [16].

Despite the importance of the problem of self-medication among university students, to authors' knowledge, only two Egyptian studies investigated that problem, one among medical students in Ain Shams University [17] and the other among nonmedical students in Suez Canal University [18].

So, the main objectives of this study were to explore: (1) prevalence of self-medication practices among university students (medical and nonmedical); (2) the most probable determinants for such practices; (3) types of medications used; and (4) sources of advice for self-medication.

## 2. Subjects and Methods

### 2.1. Study Population

A descriptive cross-sectional study was carried out in Mansoura University, Egypt, and included 1st and last year students of both medical (medicine, pharmacy, an nursing) and nonmedical (commerce and education) colleges during the academic year 2014-2015.

### 2.2. Sample Size Determination

Sample size was calculated online (https://www.dssresearch.com/). From a previous study [17] in Ain Shams University, Egypt, the prevalence of self-medication was found to be 55% and by considering the worst acceptable value as 50%; the sample size was 614 with 95% confidence level and 80% study power. The calculated sample size was multiplied by 1.5 to compensate for the design effect of the cluster sampling technique employed. Thus, the final sample size was about 900.

### 2.3. Sampling Technique

A multistage, stratified, cluster sampling technique was adopted. In the first stage, the university colleges were stratified into medical (medicine, dentistry, pharmacy, nursing, and veterinary) and nonmedical (engineering, education, physical education, commerce, agriculture, law, and arts). The sample size was distributed proportionally between both categories. In the second stage, one college or more was selected from each group by lottery method. Lastly, in each college, students were stratified into first and last academic years. From each stratum, a section (cluster) was randomly chosen. All students in the chosen clusters were included. A total of 900 students were registered in 20 chosen clusters (30–40 students in each cluster).

A total of 900 questionnaires were distributed and 800 questionnaires were returned back making a response rate of 89% due to absence of some students during the study period (3.8%), incomplete questionnaires (5%), and lack of interest in the study (2.2%).

### 2.4. Study Tool

A semistructured questionnaire was used to collect the following data: sociodemographic characteristics, for example, age, sex, residence, marital and working statuses, college, grade, and mother and father age and education; health-related questions, for example, use of any illicit (illegal) drug, care about health, current health status, last medical consultation, drugs stored at home, and treatment with or without medical supervision; self-medication practices including conditions in which the students believe that self-medication is convenient, drugs used, reasons for self-medication, source of advice, and advantage and disadvantage of self-medication; and knowledge about drugs: for example, antibiotics strengthen the immune system and some medications can be absorbed into the bloodstream through the skin.

### 2.5. Ethical Consideration

This study was approved by both the Vice Dean of the Students' Affairs and the Institutional Research Board (IRB) at Faculty of Medicine, Mansoura University. An informed verbal consent of study subjects, to participate voluntarily in the study with a full right to withdraw, was obtained with assurance of confidentiality and anonymity of the data.

### 2.6. Data Analysis

Data were entered and statistically analyzed using the Statistical Package for Social Sciences (SPSS) version 16. Qualitative data were described as numbers and percentages. Chi-square (*χ*^2^) test was used for comparison between groups. Quantitative data were described as means (SD) after testing of normality by Kolmogorov-Smirnov test. Independent sample *t*-test was used for comparison between groups. Binary stepwise logistic regression analysis was used for prediction of independent variables of self-medication. Significant predictors in the univariate analysis were entered into the regression model. Odds ratios and their 95% confidence interval were calculated. “*p* value ≤ 0.05” was considered to be statistically significant in the univariate and multivariate analyses.

## 3. Results

The mean age of students was 20 ± 0.7 years with a higher mean age of the father than the mother (53.3 and 45.5 years, resp.). Most of the students were females (78.1%) and the majority (91%) were ever married, with nearly equal distribution regarding their residence; the medical sector represented 52% while 48% were nonmedical; about 60% were in their last grade with the most frequent education of their father and mother being university and higher level (data are not shown in tables).

About 61% of students reported that their current health condition was good while only 45.1% were careful regarding their degree of care about health, and most (77.5%) stored drugs at their home pharmacy. The most frequent conditions that are suitable for self-medication from the students opinion were cold (70.1%), headache (58.9%), sore throat (35.8%), intestinal colic (32.2%), and then cramps (31%). About 59% of students mentioned that the self-medicated drugs solved the symptom (Tables 1 and 2).

The prevalence of self-medication was 62.9%. The most frequently reported cause of self-medication was “no need to visit the doctor for a minor disease” followed by “knowledge from previous experience” (73.9%  and 71.4%, resp.) and the least was “unavailability of health service,” while fear of adverse/side effects was the most frequent cause to not self-medicate. Pharmacy clerk (69.9%) and neighbors and family (62.2%) were the most frequently reported sources for self-medication compared to friends and classroom colleagues (0.6%) and old prescription (33.6%), their own decision represented 30.2%, and the Internet constituted 29.4%. As regards the academic effect on self-medication, 61.8% of students gained knowledge which made them safer for self-medication, and being more careful when self-medicating was the most frequent change in students' practice of self-medication. Regarding recent attitude of students towards self-medication, reading the package leaflet of self-medicated drug was the most frequently reported (88.8%) followed by discouraging friends and family from self-medicating (87.4%) (Table 3).

About half of the students were knowledgeable that some medications could be absorbed into the bloodstream through the skin while only 11.6% of them had knowledge regarding drug interaction (data are not shown in tables).

As regards sociodemographic characters of the studied group, there was statistically significant difference between students who self-medicate and those who do not regarding student and father ages, gender, residence, education, marital status where most of the self-medicating group were females from urban areas, medical students, and ever-married students, and lastly paternal education where most of the self-medicating group belonged to families with secondary, university, and higher education. Concerning health-related factors, there was statistically significant difference between both groups regarding their current health condition, degree of care about health, and drug storage at home pharmacy where most of the self-medicating group mentioned good health condition, being careless about their health, and storing drugs at home pharmacy. Younger age, female, medical, ever-married students, and those having home pharmacy tended to self-medicate more than their peers with significant difference between them.

Logistic regression analysis showed that being from urban area, being medical student, having good current health condition, being careless about health, and having drugs stored at home pharmacy were independently associated with the likelihood of self-medicating (OR = 1.4, 2.2, 2, 0.5, and 2.4, resp.) (Table 4).

## 4. Discussion

Prevalence of self-medication has remained common in both developing and developed countries [19, 20] and the trend is increasing among youths [21] and common among university students [22]. Socioeconomic factors, lifestyle, readily available drugs, increased medical consultation cost, time consuming clinical process, lack of nearby access to healthcare, past experiences, and extensive advertisement are some of the leading reasons for people seeking self-medication [8, 13, 23].

In this study, students had a mean age of 20 ± 0.7 years, 78.1% were female, about 60% were in their last grade, 52% belonged to the medical sector, and 77.5% of students stored drugs at their home pharmacy. Similarly in Karachi, the mean age of university students who participated in this study was 21 ± 1.8 years. Amongst them 51.6% were medical students while 48.4% were nonmedical. There were 41.1% males and 58.9% females. They obtained drugs mostly from a pharmacy (64.6%) or/and stocks kept at home (64.4%) or from friends (9.7%) [3].

In our study, the most frequent conditions in which the student self-medicated were cold, headache, sore throat, intestinal colic, and lastly cramps. About 59% of students mentioned that the self-medicated drugs solved the symptom.

Similarly, the overall reasons for self-medication among university students from the city of Rio Grande, Brazil, were headache, cold, sore throat, fever, menstrual cramps, muscle pain, cough, and heartburn as well as stomachache, nausea, vomit, allergy, and intestinal colic [24]. In Karachi, the most common symptoms were headaches, fever, and flu-like symptoms [3]. In Abbottabad, Pakistan, the most common symptom was “storage of medicines for multipurposes” (50.8%). This was followed by occasional pain, common infections, and cough/cold. About 67.2% of respondents were successfully treated by self-medication, while 33.7% experienced more severity in disease conditions [25].

In Nepal, fever, headaches, and cough were the predominant indications for self-medication among undergraduate pharmacy students followed by diarrhoea, cold, acidity, and pain condition. This was in accordance with that of the Association of European Self-Medication Industry which had enlisted pain, allergy, colds, sore throat, cough, and diarrhoea as common diseases for preferring self-medication [26].

This study showed that pharmacy clerk, neighbors, and family were the most frequently reported sources for self-medication and lastly Internet. In Nepal, friends and family, chemist, and Internet were registered as top three sources of information by the undergraduate pharmacy students [26].

The prevalence of self-medication in this study was 62.9% with significantly higher prevalence in the medical sector than the nonmedical one (72.4%  and 52.6%, resp.). Our finding was in accordance with that study in Karachi, where it was found to be 76% with no significant difference between medical and nonmedical students [3].

A higher prevalence of self-medication was reported by some studies. It was found to be 95.5% in Abbottabad, Pakistan, with statistically nonsignificant (*p* = 0.099) difference between health and nonhealth students [25]. In the city of Rio Grande, Brazil, 86.4% of university students reported self-medication, 58% were healthcare students, and 61% were first-year students [24]. It was found to be 88% in Croatia [27], 94% in Hong Kong [28], 98% in Palestine [8], and 92.3% in Slovenia [9].

This could be explained by the fact that nonmedical students also consider themselves to be as knowledgeable about medicines as medical students, or it just may be that university students both medical and nonmedical do not care much about the implication of such behavior and thus do not hesitate to indulge in such activities [3].

A lower prevalence of self-medication was reported by some studies. In a Brazilian study, 57.7% of university students in the city of Recife declared not to self-medicate [29]. Also, it was found to be up to 45% in Turkey [30]. It may be explained that healthcare-related education of students led to more responsible self-medication [9].

The most frequently reported causes of self-medication were “no need to visit the doctor for a minor disease” and the least was “unavailability of health service,” while “fear of adverse/side effects” was the most frequent cause for not self-medicating. In accordance with our results, in that study in Brazil, the first reason cited by university students was “I have already experienced the symptom and know what to take” (57.2%) [24]. This may reflect a usual behavior and the repeated use of an old prescription. Storage of medications at home with free access and easy visualization of the drugs is a risk factor for self-medication [31]. Receiving advice mainly from the family (53.1%) about self-medication and the reuse of old prescription (40.4%) contribute to the risk posed by home pharmacies [24]. This suggests an easy access to medications and a culturally inherited acceptance of self-medication [32]. Further explanations for self-medication cited by students in this study included “there is no need to see a doctor because of a simple disease” and “quick relief.” These explanations could be supported also by the existence of home pharmacy [24].

In Nepal, it is to be noted that 36.47% perceived self-medication as unacceptable practice while 47.64% said it was an acceptable practice. When asked about the reason where practicing self-medication would be considered unacceptable, seldom effectiveness (12.35%), adverse reaction (16.47%), unsafe (37.46%), and likely side effects (18.23%) were the main reasons among undergraduate pharmacy students [26]. In Karachi, the most common factors that led to self-medication were “previous experience with similar symptoms” (50.1%) and self-perception of “trivial nature of the problem” (48.3%) [3].

However, in Abbottabad, Pakistan, the most common factor responsible for self-medication was “low severity of disease” (45.7%) and the second most common factor was “told by doctor verbally” (44.9%). Other causes of self-medication were lack of access and time and financial issues [25].

In contradiction with our study, in Southwestern Nigeria, the issue of long waiting queues at clinics or hospitals was raised by 59% of university students as one of the reasons for seeking self-care in order to meet up with their tight lecture schedule [33].

About 62% of students in this study gained knowledge which made them safer for self-medication; being more careful when self-medicating and reading the package leaflet of self-medicated drug were the most frequent change in practice and recent attitude of students towards self-medication, respectively.

Similar results were obtained in Brazil where a high percentage of university students replied that they discouraged their friends and relatives from self-medication. Larger number of healthcare students discouraged their friends and relatives from self-medication (85.8 versus 76.6%, resp.; *p* < 0.001) [24].

In this study, younger age, female, medical, and ever-married students and those who have home pharmacy tended to self-medicate more than their peers with significant difference between them. Similarly, in Southwestern Nigeria, female students exhibited higher prevalence of self-medication than their male counterpart. At the undergraduate levels, the prevalence of self-medication increased marginally from 1st year to 5th year students. Self-medication was significantly associated with age, gender, and students' level in the university at *p* < 0.001 [33].

In Brazil, age, male sex, employment, having a partner, and having children were significantly associated with self-medication among university students in the bivariate analysis. In the healthcare program, existence of a home pharmacy was significantly associated with self-medication. The same was for last year students from healthcare versus nonhealthcare programs (*p* = 0.01). Lastly, poor medication knowledge was significantly associated with less self-medication while being a first or last year student did not affect the outcome [24].

However, in Karachi, there was no significant difference between self-medication practices of medical and nonmedical students, males and females, or the year of study. Self-medication rates were not significantly lower in students aware of its harmful effects (*p* = 0.21) [3]. Similarly, in Abbottabad, Pakistan, there was nonsignificant difference in self-medication between the two genders of university students [25].

Logistic regression analysis showed that being from urban area, being medical student, having good current health condition, being careless about health, and having drugs stored at home pharmacy were independent predictors for self-medicating (OR = 1.4, 2.2, 2, 0.5, and 2.4, resp.).

In Brazil, sex, having children, illicit drug use, and having a home pharmacy were statistically associated with self-medication among university students in the multivariate analysis. Poor medication knowledge was significantly associated with less self-medication [24]. Storage of medications at home with free access and easy visualization of drugs is a risk factor for self-medication [31].

## 5. Limitations of This Study

This study suffers some limitations. The questionnaire was self-reported which could have led to underreporting of self-medication practices.

The structure of questionnaires could affect prevalence estimates; longer questionnaires could result in a higher prevalence of self-reported self-medication, whereas shorter questionnaires with open questions could result in a lower prevalence of self-reported self-medication within the same population [34].

## 6. Conclusion

In conclusion, our findings demonstrated that self-medication is prevalent among Mansoura University students. Being from urban area, being medical student, having good current health condition, being careless about health, and having drugs stored at home pharmacy are independent risk factors for self-medication among them. Proper counseling and public health education together with strict regulations on drug advertisement and supply would be successful interventions.

## 7. Recommendations

The prevalence of self-medication practices is unexpectedly high whether among medical or nonmedical students. This could be attributed to the long waiting queues at clinics or hospitals and country poor regulations and relaxed laws, which manifest as weak control over both sale of medicines from pharmacy stores without doctor's prescription and advertisement which affects the youth decision to self-medicate. Suggested solutions to combat this problem could include the following approaches: (i) health professionals should actively participate through counseling and public health education about problems that may arise from inappropriate use of medications, (ii) health facilities should be available to each individual with much less difficulties, and (iii) strict rules regarding pharmaceutical advertising and supply of medications without pharmacies' prescription should be laid down.

## Figures and Tables

**Table 1:** Health-related information of students.

Information	*N*	%
Current health condition		
Excellent	84	10.5
Good	486	60.8
Fair	230	29.2
Degree of care about health		
Careful	361	45.1
Careless	439	54.9
Drug storage at home pharmacy	620	77.5

Total	800	

**Table 2:** Reasons for self-medication among medical and nonmedical students.

System	*N*	%
*Respiratory*		
Cold	561	70.1
Sore throat	286	35.8
Cough	227	28.4
*Gastrointestinal*		
Intestinal colic	258	32.2
Diarrhea	225	28.1
Constipation	201	25.1
Heartburn	172	21.5
Vomiting	154	19.2
Poor digestion	104	13.0
Liver problems	39	4.9
*Pain & musculoskeletal*		
Cramps	248	31.0
Toothache	164	20.5
Muscle pain	64	8.0
Earache	45	5.6
*Neurological *		
Headache	471	58.9
Sickness	125	15.6
Sleep disorders	64	8.0
Lack of attention	28	3.5
Anxiety	20	2.5
Tiredness	17	2.1
*Others *		
Allergy	60	7.5
Fever	41	5.1
Urinary tract infection	24	3.0
Weight loss	23	2.9
Skin rash	22	2.8

The self-medicated drugs solved the symptom	**473**	**59.1**

Total	800	

**Table 3:** Explanations for self-medication.

Prevalence	*N*	%
Self-medication	503	62.9
No self-medication	297	37.1
*Causes of self-medication (503)* ^*#*^		
No need to visit the doctor for a minor disease	372	73.9
Knowledge from previous experience	359	71.4
The doctor will prescribe me the same drug	155	30.8
Time and money saving	119	23.7
Fast relief	66	13.1
Chance to have experience	36	7.2
Absence of trust in health services	27	5.4
Unavailability of health service	15	2.9
*Causes for not self-medicating (297)* ^*#*^		
Fear of adverse/ side effects	124	41.8
Lack of knowledge & experience	73	24.6
Lack of confidence	52	17.5
It is injurious to health	31	10.4
Prior bad experience with self-medication	17	5.7
*Sources for self-medication (503)* ^*#*^		
Pharmacy clerk	352	69.9
Neighbors & family	313	62.2
Friends & classroom colleagues	204	40.6
Old prescription	169	33.6
My decision	152	30.2
Internet	148	29.4
Books & magazine	24	4.8
*Academic effect on self-medication:*		
(i) knowledge gained in college makes students safer for self-medication	**494**	**61.8**
(ii) Changes observed in practice of self-medication as the students acquire more academic knowledge^#^		
More careful when I self-medicate	313	39.1
More concerned about adverse effects/side effects/interactions	180	22.5
More confident self-medicating	133	16.6
No change	86	10.8
I would rather use a prescription	75	9.4
(iii) Recent attitude towards self-medication^#^		
Reading the package leaflet of self-medicated drugs	710	88.8
I discourage friends and family from self-medicating	699	87.4
Believe what they read in the leaflet of medicinal products	698	87.2
I encourage friends and family to self-medicate	101	12.6

Total	800	

^#^Categories are not mutually exclusive.

**Table 4:** Adjusted and crude analysis of the variables associated with self-medication.

	Self-medicating *N* = 503	Not self-medicating *N* = 297	*p* value	Univariate analysis OR (CI)	Multivariate analysis OR (CI)
*Sociodemographic characters*					
Student age	19.9 ± 0.7	20.1 ± 0.7	0.000		
Mother age	45.7 ± 5.1	45.1 ± 5.1	0.083		
Father age	53.9 ± 12.2	52.1 ± 5.9	0.02		
Sex					
Female	411 (65.8)	214 (34.2)	0.002	1.7 (1.2–2.4)	
Male (R)	92 (52.6)	83 (47.4)			
Residence					
Urban	274 (68.2)	128 (31.8)	0.002	1.6 (1.2–2.1)	1.4 (1.1–1.9)
Rural (R)	229 (57.5)	169 (42.5)			
Education					
Medical	301 (72.4)	115 (27.6)	0.000	2.4 (1.8–3.2)	2.2 (1.6–2.9)
Nonmedical (R)	202 (52.6)	182 (47.4)			
Father education					
Illiterate (R)	27 (46.6)	31 (53.4)			
Primary and preparatory	57 (54.8)	47 (45.2)	0.31	1.4 (0.7–2.8)	
Secondary	132 (67.3)	64 (32.7)	0.004	2.4 (1.3–4.5)	
University and higher	287 (64.9)	155 (35.1)	0.006	2.1 (1.2–3.8)	
Mother education					
Illiterate (R)	42 (47.2)	47 (52.8)	0.16		
Primary and preparatory	52 (57.8)	38 (42.2)		1.5 (0.8–2.9)	
Secondary	149 (62.6)	89 (37.4)	0.011	1.9 (1.1–3.2)	
University and higher	260 (67.9)	123 (32.1)	0.0002	2.4 (1.4–3.9)	
Marital status					
Never married	35 (48.6)	37 (51.4)	0.009	0.5 (0.32–0.86)	
Ever married (R)	468 (64.3)	260 (35.7)			
Grades					
1st grades	197 (61.0)	126 (39.0)	0.37	0.9 (0.65–1.17)	
Final grades (R)	306 (64.2)	171 (35.8)			

*Health-related factors*					
Current health condition					
Excellent	46 (54.8)	38 (45.2)	0.9	1 (0.6–1.6)	1.3 (0.74–2.3)
Good	329 (67.7)	157 (32.3)	0.002	1.7 (1.2–2.3)	2.0 (1.4–2.9)
Fair (R)	128 (55.7)	102 (44.3)			
Degree of care about health					
Careful	204 (56.5)	157 (43.5)	0.001	0.61 (0.5–0.8)	0.5 (0.4–0.9)
Careless	299 (68.1)	140 (31.9)			
Drug storage at home pharmacy					
No	78 (49.7)	79 (50.3)	0.58	1.28 (0.5–3.4)	1.3 (0.5–3.4)
Yes	415 (66.9)	205 (33.1)	0.02	2.6 (1.1–6.6)	2.4 (1.0–5.9)
I do not know (R)	10 (43.5)	13 (56.5)			

Model *χ*^2^ = 82%, *p* < 0.0001,   % correctly predicted = 67.8%, and constant = −0.974.
